# Subtle Sonographic Signs of Disseminated Tuberculosis: A Case Report and Narrative Literature Review

**DOI:** 10.4269/ajtmh.24-0100

**Published:** 2024-07-16

**Authors:** Daniel Z. Hodson, Tapiwa Kumwenda, Claudia Wallrauch, Ethel Rambiki, Christopher Tymchuk, Francesco Taccari, Tom Heller

**Affiliations:** ^1^ Division of Internal Medicine—Pediatrics, David Geffen School of Medicine at UCLA, Los Angeles, California;; ^2^ Lighthouse Clinic Trust, Lilongwe, Malawi;; ^3^ LMU University Hospital Munich, 4th Medical Department, Division of Infectious Diseases and Tropical Medicine, Munich, Germany;; ^4^ Division of Infectious Diseases, David Geffen School of Medicine at UCLA, Los Angeles, California;; ^5^ UOC Malattie Infettive - Dipartimento di Scienze, Mediche e Chirurgiche - Fondazione Policlinico Universitario A. Gemelli IRCCS, Rome, Italy;; ^6^ International Training and Education Center for Health, University of Washington, Seattle, Washington

## Abstract

Miliary tuberculosis is a form of disseminated tuberculosis that can be difficult to detect when the classic pattern is absent on chest radiograph and advanced cross-sectional imaging is not readily available. While the focused assessment with sonography for HIV-associated tuberculosis (FASH) protocol for extrapulmonary tuberculosis emphasizes easy-to-teach findings, experienced sonographers may detect additional, subtler signs that can aid in diagnosis. We report a case of a 20-year-old man with miliary tuberculosis diagnosed on computed tomography of the chest. We describe subtle sonographic signs of disseminated tuberculosis including subpleural irregularities and comet-tail artifacts, a bright liver pattern, peritoneal nodules, and a nonspecific sponge spleen pattern. We then discuss important differential diagnoses for each finding. Knowledge of subtle sonographic signs outside of the FASH protocol can aid clinicians in detecting disseminated tuberculosis, including the miliary form, when advanced imaging may not be available.

## INTRODUCTION

Mycobacterial spread in disseminated tuberculosis (TB) results from “massive lymphohematogenous dissemination” seeding multiple organs, and disseminated TB may occur with primary infection or reactivation.^1^^,^^2^ Miliary TB is a specific manifestation of disseminated TB named for the similarity between its characteristic appearance on chest imaging and the appearance of millet seeds on the stalk.^1^^,^^2^

Diagnosis of miliary TB proves challenging. Symptoms and physical signs are nonspecific, save for the rare finding of choroidal tubercles on fundoscopy.^2^ Pulmonary involvement may be absent at the time of presentation. For example, one autopsy-based study found only 48% of patients presented with pulmonary symptoms and only 86% had evidence of pulmonary involvement on pathologic examination.^3^ Sputum samples in miliary TB are usually acid-fast bacilli smear negative,^4^ and GeneXpert^®^ MTB/RIF (Cepheid, Sunnyvale, CA) systems (Xpert) may exhibit decreased sensitivity in smear-negative sputum samples.^5^^,^^6^ Even cases with pulmonary involvement may go undetected in settings without access to cross-sectional imaging because the characteristic miliary pattern on plain chest radiograph may be absent in ∼50% of cases.^1^^,^^2^^,^^7^ For example, another seminal autopsy-based review from the pre–computed tomography era found that only 25% of cases were diagnosed before death despite pathologic evidence of pulmonary involvement in 71% of cases.^8^ Purified protein derivative, interferon-gamma assays, and microbiological testing of body fluid suffer from suboptimal sensitivity in miliary TB,^9^ and even Xpert systems may show decreased sensitivity for extrapulmonary specimens.^10^^,^^11^

Thus, cross-sectional chest imaging and/or invasive biopsy with pathologic review can be needed for definitive diagnosis of miliary TB, but these options may not be readily available in under-resourced settings. Point-of-care bedside ultrasound (POCUS) can aid in identification of disseminated and miliary TB. Although the focused assessment with sonography for HIV-associated tuberculosis (FASH) protocol for extrapulmonary TB has been well described and is widely used,^12^^–^^15^ its main focus remains easy-to-teach and easy-to-detect findings. Additional and under-recognized sonographic signs of miliary TB can be detected by experienced sonographers. We describe a case of miliary TB to highlight characteristic sonographic findings, discuss the main differential diagnoses of these findings, and review the pertinent literature.

## CASE PRESENTATION

**Figure 1.**
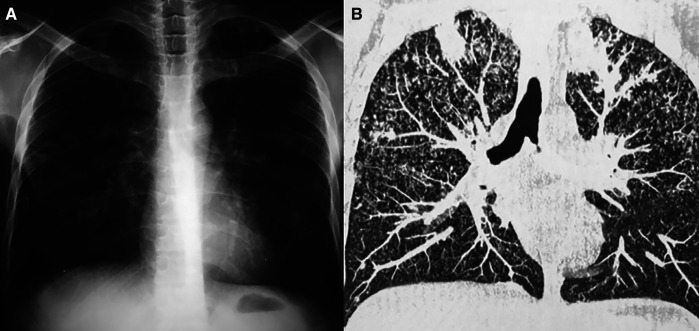
Chest radiograph (**A**) showed only nonspecific opacities. Computed tomography (**B**) revealed numerous cavitations along with the classic miliary pattern.

**Figure 2.**
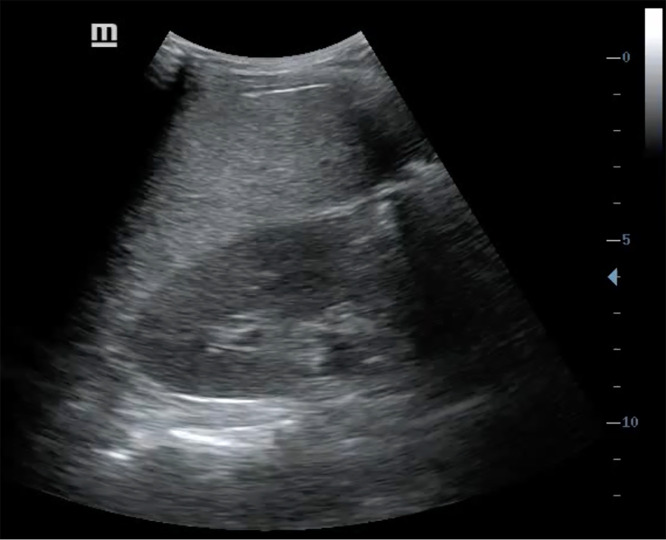
Ultrasound of the right upper quadrant revealed a diffusely hyperechoic “bright liver” pattern without focal lesions.

**Figure 3.**
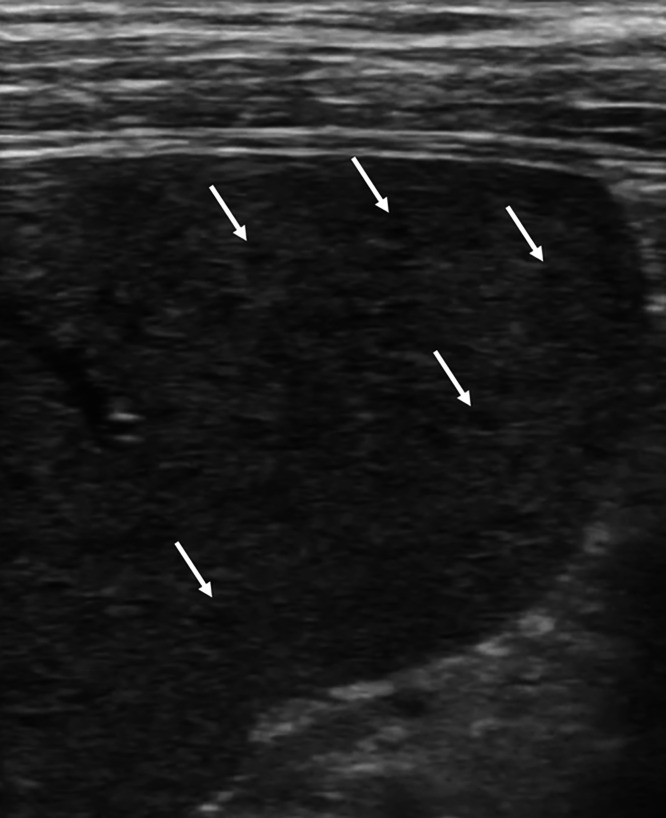
Ultrasound of the spleen revealed numerous 1- to 2-mm hypoechoic lesions (arrows) in a “sponge-like” pattern.

**Figure 4.**
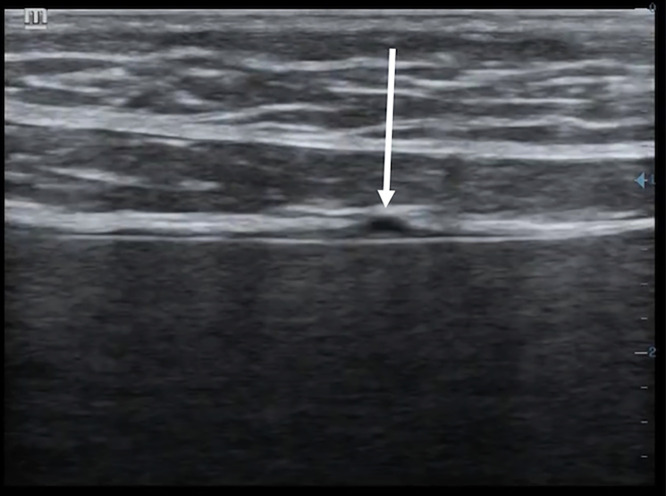
Ultrasound of the peritoneum superficial to the liver revealed multiple, hypoechoic <3-mm micronodules.

**Figure 5.**
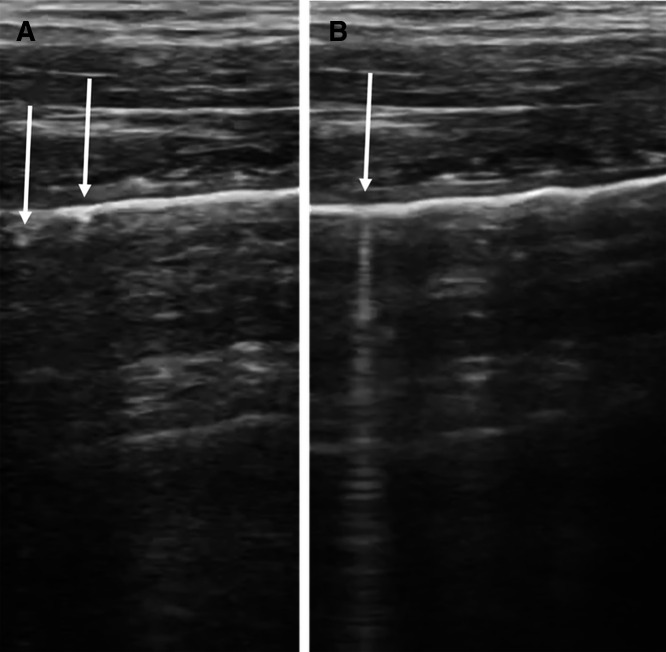
The visceral-parietal pleural interface was irregular with hyperechoic subpleural microlesions (**A**) and innumerable vertical, comet-tail artifacts (**B**). An A-line pattern was also still present.

A 20-year-old HIV-negative man was referred in 2023 with concern for TB to Lighthouse Clinic, a referral HIV/TB treatment center in Lilongwe, Malawi. He complained of weakness, dry cough, night sweats, and weight loss. His physical examination was notable only for a weight of 62 kg with a body mass index of 19.4 kg/m^2^. Complete blood count was notable for hemoglobin 10.3 g/dL. Liver chemistries showed a predominately cholestatic pattern with alkaline phosphatase 668.0 U/L (reference range [RR] 42.0–98.0), gamma-glutamyl transferase 766.4 U/L (RR 9.0–78.0), aspartate aminotransferase 78.3 U/L (RR ≤35.0), alanine aminotransferase 57.9 U/L (RR ≤45.0), and total bilirubin 0.63 mg/dL (RR ≤2.0). Serum creatinine was normal. GeneXpert^®^ MTB/RIF performed on expectorated sputum was negative. Chest radiograph (Figure 1A) showed nonspecific bilateral opacities. A FASH scan performed using low-frequency convex and high-frequency linear probes was negative. On more detailed assessment, the liver was markedly hyperechoic compared with the adjacent kidney (Figure 2 and Supplemental File 1). Focal liver lesions were absent. Examination of the spleen revealed innumerable, 1- to 2-mm hypoechoic lesions (Figure 3 and Supplemental File 2). Multiple hypoechoic micronodules were also found in the peritoneum between the liver and abdominal wall (Figure 4 and Supplemental File 3). Examination of the visceral-parietal pleural interface (VPPI) revealed multiple, echogenic foci associated with vertical, comet-tail artifacts (Figure 5 and Supplemental File 4). Computed tomography of the chest revealed the classic miliary pattern and numerous cavitations (Figure 1B). The patient was initiated on first-line TB treatment with rifampin, isoniazid, pyrazinamide, and ethambutol according to Malawi national guidelines^16^ and was followed at monthly, in-person appointments for the 6 months of treatment. Repeat sonographic assessment is not routinely indicated and was not performed in this case. He completed treatment, his weight increased to 67 kg, and he was doing well when contacted for phone follow-up 7 months after diagnosis.

## DISCUSSION

We present a case of miliary TB with suggestive ultrasound findings in the lung, liver, and peritoneum. Heller and colleagues previously introduced the FASH protocol in this journal,^12^ and the protocol has been incorporated into daily practice in multiple under-resourced settings with high incidence of HIV and TB.^15^^,^^16^ The FASH allows bedside detection of focal findings that increase the probability of TB; these findings include pericardial effusions, unilateral pleural effusions, enlarged abdominal lymph nodes, splenic micro-abscesses, and ascites.^12^^,^^13^^,^^17^ Our case highlights additional and more subtle findings in miliary TB. Like the FASH findings, these signs are suggestive of disseminated TB (miliary or otherwise) but must also be interpreted in the context of the overall clinical presentation.

Liver involvement in disseminated TB can present as hypoechoic mycobacterial micro-abscesses.^13^ In our patient, we documented the liver to be diffusely *hyperechoic* relative to the adjacent kidney and detected a cholestatic pattern from the liver chemistries, suggesting granulomatous hepatitis. Prominent liver involvement in miliary TB has been noted for more than 100 years,^18^ and granulomatous hepatitis has even been included in the definition of miliary spread.^3^^,^^8^ Case reports have previously described the “bright liver” pattern in disseminated TB.^19^^–^^22^ Proposed histopathological explanations for this pattern include “acoustic mismatch” between the TB micronodules and surrounding liver parenchyma^19^; granulomas themselves in TB and other granulomatous disease^23^; and “acoustic impedance” created by the interface of fibrosis and exudate amidst healthy parenchyma.^24^ The differential for the bright liver pattern includes several infectious and noninfectious etiologies (Table 1). In addition, TB infiltration of the liver may also lead to hyperechoic focal lesions.^25^^–^^27^ A focal, hyperechoic lesion may reflect tubercles without caseating necrosis.^26^

**Table 1:** Key differential diagnoses for sonographic findings of miliary tuberculosis

Sonographic Window	Sonographic Findings	Key Differential Diagnoses
Lung	Irregular pleura with subpleural consolidations Diffuse comet-tail or B-line artifacts	Nontuberculous mycobacteria Viral pneumonia^53^^–^^57^ Disseminated fungal infections^58^ Interstitial lung disease^59^ Acute respiratory distress syndrome^60^
Liver	Bright liver pattern	Nontuberculous mycobacteria^20^^,^^23^ Other bacterial infections^23^ Disseminated fungal infections^24^ Hepatic steatosis and cirrhosis^61^ Lymphoma^62^ Sarcoidosis^63^^–^^65^
Spleen	Sponge pattern	Nontuberculous mycobacteria^28^ Other bacterial infections^28^^,^^66^ Lymphoma^28^^,^^67^ Disseminated fungal infection^28^ Kaposi sarcoma^28^^,^^68^ Multicentric Castleman disease^28^ HIV without superimposed opportunistic infection^28^
Abdomen^†^	Peritoneal thickening and nodules Omental and mesenteric thickening or caking Intestinal thickening Pelvis masses	Nontuberculous mycobacterial infection^69^ Ovarian cancer^36^^,^^40^^,^^41^^,^^43^ General peritoneal carcinomatosis Crohn disease^33^^,^^38^^,^^70^ Mesothelioma^71^ Bacterial infections (e.g., *Yersinia, Actinomyces*) Parasitic infection (e.g., *Entamoeba*) Lymphoma Sarcoidosis

FASH = focused assessment with sonography for HIV-associated tuberculosis.

^†^ The common findings of ascites^9^^,^^22^^,^^29^^–^^47^^,^^72^^,^^73^ and lymphadenopathy,^22^^,^^30^^–^^35^^,^^37^^,^^40^^,^ ^42^^,^^43^^,^^45^^,^^46^^,^^72^^,^^73^ which are already included in the FASH protocol, are not included here.

In the FASH, the spleen is assessed for micro-abscesses, which are usually larger than 5 mm.^12^^,^^13^ In our case, we found only 1- to 2-mm lesions suggesting a “sponge pattern,” which likely represents a hyperplastic white pulp of the spleen.^28^ This is seen in a variety of disseminated infections and is not necessarily suggestive of TB (Table 1).

The abdomen is examined in the FASH for enlarged periportal/periaortic lymph nodes and ascites as evidence of abdominal TB, but the intestines, mesentery, and peritoneum are not directly assessed.^12^^,^^13^ In our case, we found hypoechoic micronodules in the peritoneum. Sonographic evidence of TB in the abdomen has been described for over 45 years.^29^ Ascites (often with debris and/or thin, fibrous septations) and lymphadenopathy are well-recognized findings; thus, these are included in the FASH protocol.^12^^,^^13^ Peritoneal thickening^9^^,^^30^^–^^36^ and peritoneal nodules^30^^,^^32^^,^^33^^,^^35^^,^^37^^,^^38^ have been described previously. In our experience, peritoneal micronodules appear as multiple, discrete, hypoechoic nodules <5 mm located immediately superficial to the hyperechoic liver capsule. These micronodules are best detected by scanning with a high-frequency linear probe in the epigastrium for the left hepatic lobe and in the right inferior intercostal spaces for the right hepatic lobe. Endoscopic or surgical visualization of the peritoneum may reveal classic miliary nodules on the peritoneum,^9^^,^^34^^,^^38^^–^^40^ and peritoneal biopsy reveals granulomas with or without caseating necrosis.^9^^,^^30^^,^^38^^–^^41^ Other described sonographic findings of abdominal TB include omental thickening or “caking,”^22^^,^^31^^,^^32^^,^^35^^,^^36^^,^^42^^–^^44^ intestinal thickening,^22^^,^^30^^–^^33^^,^^38^^,^^45^^,^^46^ adherent bowel loops,^30^^,^^37^^,^^42^^,^^45^^,^^47^ mesenteric thickening,^31^^,^^32^^,^^34^^,^^45^ and pelvic masses.^36^^,^^43^^,^^46^ Two additional sonographic signs are the “club sandwich sign” created by exudate floating in ascites between bowel loops^30^^,^^32^ and the “stellate sign” created by matted bowel loops radiating around a thickened mesentery.^32^ The differential for these findings includes both infectious and noninfectious etiologies (Table 1). The sonographic findings are dynamic, and both ascites and mesenteric thickening can resolve with treatment.^30^^,^^45^ Elevated CA-125 may confuse the diagnostic picture by increasing suspicion for ovarian cancer,^40^^,^^41^^,^^43^ so this test has even been suggested as a biomarker for TB.^48^

The FASH detects pleural effusions, a well-known finding suggesting pleural TB, but the VPPI and lung parenchyma are not directly examined.^12^^,^^13^ In our case, we found an irregular VPPI with innumerable comet-tail artifacts. A South African case series previously described miliary lesions presenting as granular pleural irregularities and vertical artifacts emerging from the VPPI.^49^ Subpleural nodules may be the most sensitive sonographic sign for TB in general.^50^ The sonographic signs are analogous to the findings on chest imaging and histopathology. The gross pathological appearance of the classic miliary lesion has been described as “the millet-sized tubercle, approximately 1–2 mm in diameter.”^3^ On chest imaging, this classically manifests as diffuse, micronodular opacities <3 mm,^7^ but pathological changes may be subtle or absent on chest radiograph and require computed tomography to visualize fully.^51^^,^^52^ The differential for these findings again includes both infectious and noninfectious etiologies (Table 1).

In summary, we report subtle sonographic clues from various organs that can raise suspicion for disseminated TB, including its miliary form. Although most associated with HIV, disseminated TB can occur in HIV-negative individuals, such as our patient. Accurate diagnosis is critical, as misdiagnosis and delayed initiation of treatment can lead to a detrimental outcome. By incorporating POCUS into the initial assessment and integrating sonographic signs into the overall clinical picture, clinicians can narrow the differential diagnosis more quickly and thus expedite the next steps in workup and management.

## Supplemental Materials

10.4269/ajtmh.24-0100 Supplemental Materials

10.4269/ajtmh.24-0100 Supplemental Materials

10.4269/ajtmh.24-0100 Supplemental Materials

10.4269/ajtmh.24-0100 Supplemental Materials
